# Uniform Compliments Invite Criticism on Dublin Hospitals

**Published:** 1915-10-09

**Authors:** 


### Uniform Compliments Invite Criticism on Dublin Hospitals.

In their latest Annual Report on those of the Dublin hospitals which receive grants from Parliament, the Board of Superintendence finds much matter for congratulation and commendation, and little opportunity for criticism or suggestion. Of Steevens' Hospital it is recorded that there is "nothing but satisfaction to express." Note is taken of the introduction of the "open-air" method of treatment, of the repainting of the fever wards, of the introduction of tiled lavatories, and of a new drying system in the laundries. At the Lock Hospital the Board found affairs so satisfactory that they "do not see how the governing body could do more for the institution than is being done." The matron and the registrar are both specially commended, and appreciation is expressed of the bath accommodation and of the installation of an electric lighting plant. The Meath Hospital is burdened with a heavy debt, but manages to keep abreast of the times; among the developments of the year are new balconies which make provision for escape in the case of fire, and are utilised also for convalescent patients. Everything was "in excellent working order" at the Cork Street Fever Hospital, and every detail of administration was carefully scrutinised. The Rotunda Lying-in Hospital has, of course, a renown far beyond the city where it is established, and the inspectors tell us that the hospital is distinguished by an excellent routine which works with the most admirable results; these results are the more noteworthy seeing that "cleanliness and routine are not strong points in Ireland." The Coombe Lying-in Hospital receives similar praise and is recognised as doing good work both in the professional and in the educational spheres. At the Royal Victoria Eye and Ear Hospital everything speaks of growth and betterment, which the Royal Hospital for Incurables showed every department working well and no indication of extravagance or of waste. The Hardwicke Hospital, which is the only fever hospital on the north side of Dublin, has long given anxiety to the governing body. The building is old and requires extensive modification to bring it up to modern demands. Suitable plans have been prepared and indicate a necessary expenditure of some £12,000, and a grant in aid has been suggested to the Government. Unhappily, the times in which we live are not favourable to Government contributions or to private benefactions. Altogether the reports from the Board show that a high level of hospital efficiency is cultivated in Dublin, though in the midst of so many compliments the thought does arise whether the inspectors might not have been more helpful had they cultivated a more critical eye. It is quite right that good points should be observed and emphasised, and it would be a poor performance merely to seek out defects. But one of the main objects of inspection is to bring new and expert minds into contact with the existing facts, in the hope that from such experience suggestions for improvements may arise. Compliment and censure have their places, but helpful proposals are better than either.

